# Antifertility activity of aqueous ethanolic extract of *Hymenocardia acida* stem bark in female rats

**Published:** 2011

**Authors:** Abu Adakole Hyacinth, Uchendu Chukwuka Nwocha

**Affiliations:** 1 Department of Veterinary Physiology, Pharmacology and Biochemistry, College of Veterinary Medicine, University of Agriculture, P.M.B, 2373, Makurdi, Nigeria.; 2 Department of Veterinary Physiology and Pharmacology, Faculty of Veterinary Medicine, University of Nigeria, Nsukka, Nigeria.

**Keywords:** *Albino rats*, *Antifertility*, *Hymenocardia acida*, *Reproduction*

## Abstract

**Background:**

*Hymenocardia acida* is traditionally used in African herbal medicine and has numerous therapeutic benefits. But little is known about its potentially negative effects on pregnant women.

**Objective:**

The aim of the present study was to evaluate the antifertility effect of aqueous ethanolic extract of Hymenocardia acida stem bark in female Wistar rats.

**Materials and Methods:**

Four groups of rats were administered orally aqueous ethanolic extract of *Hymenocardia acida* at doses of 100, 200 and 400 mg/kg body weight daily for 19 days. The control group received distilled water. On day 20 of gestation, each rat was laparatomised and number of corpora lutea of pregnancy, number of live fetuses as well as the postcoitum fertility index, weights of the foetuses and placentae were determined.

**Results:**

Oral administration of the extract from days 1 to 19 of gestation showed reduction (p<0.05) in the number of corpora lutea of pregnancy and number of live fetuses. Weights of fetuses of extract treated female rats were also smaller (p<0.05) compared with the control. Anti-implantation activity of the treatment groups were 41.4%, 48.3% and 51.7% for groups II to IV respectively, whereas antifertility activity of the groups was found to be 40%, 60% and 60% in the same order.

**Conclusion:**

The results suggest that aqueous ethanolic extract of Hymenocardia acida stem bark could induce negative effects on reproductive functions in female albino rats.

## Introduction

There is an increasing trend in the use of medicinal plants, botanicals or herbal preparations particularly in developing countries where these products are readily available. Several animal studies have revealed anti zygotic, blastocytotoxic, anti-implantation and abortifacient properties of water and organic solvent extracts of many commonly used medicinal plants, sometimes in dose dependent manner. In an investigation of antifertility property of a triterpenoid glycoside isolated from *Dalbergia* saxatilis in female rats, a decrease in maternal body weights and inhibition of conception were observed (1). Similar observations on antifertility, antiimplantation or pregnancy interceptory properties suggestive of anovulatory, antiprogesterogenic or estrogenic effects have been made on extracts of *Calotropis gigantea* (2) and *Morinda citrifolia* (3). 

However, other animal studies showed none inhibitory effects of plant extracts on female reproductive functions. For example, *Carapa guianensis* seed oil administered orally during the period of organogenesis failed to impair implantation and induce the death of foetuses (4). 

Sensitivity of experimental animals, dose of extract used, period and route of administration as well as physiological or pharmacological mechanisms are some of the factors affecting the implantation process.

*Hymenocardia acida* (Tul.) is a small browse tree or shrub with palatable foliage, widely distributed within the savanna region of Nigeria. It is called “Enache” by Idoma people of North Central Nigeria while the local or vernacular name among the Hausas in Nigeria is “Janyaro”. All parts of the plant are useful as remedies for many ailments. The powdered roots or stem bark decoction are used to treat fever, jaundice, muscular pains, diarrhoea, dysentery and sexual incapacity in males. It is commonly used as an agent for female genital hygiene. Experimental studies have also confirmed some of the claimed efficacy of this medicinal plant by the native healers. For example, crude extracts of the plant have been reported to possess anti-tumor, anti-HIV and anti-inflammatory (5), anti-sickling (6), anti-ulcer (7) and anti-diarrhoeal (8) activities. The leaf infusion is also used in the treatment of urinary tract infections (5) and as topical applications for skin diseases in Nigeria. Among the Idoma and Igede people of North Central Nigeria, the decoction of root and stem bark is used in the treatment of diabetes (9). Also there have been reports of *in vitro* antitrypanosomal efficacy of leaf (10) and root bark (11) as well as antiplasmodial (12) activities of *Hymenocardia acida.* Despite the popular use and numerous therapeutic benefits of *Hymenocardia acida*, little is known about the effect of crude extract of *H. acida* on reproduction. The aim of the present study was to evaluate the effect of aqueous ethanolic extract of *H. acida* stem bark on fertility of female Wistar rats. 

## Materials and methods

**Plant material**

The stem bark of *Hymenocardia acida* was collected within the premises of University of Agriculture, Makurdi and authenticated by Mr. Patrick Ekwuno of College of Forestry, University of Agriculture, Makurdi, Nigeria Voucher specimen was deposited at the College herbarium. 

**Preparation of crude extract**

The stem bark was washed, air dried at room temperature for one week, pulverized and stored in air-tight container until required. As previously described (13), one hundred gram of powdered material was soaked in 500 ml of 70% ethanol and stirred intermittently for 48 hours at room temperature. The material was filtered using sterile cotton wool and Whatman (No. 1) filter paper; the residue was resuspended in the same amount of solvent and then filtered three more times. The pooled filtrates obtained were dried at room temperature under the electric fan. The extracts were stored in air-tight containers at 4^o^C until needed. 

**Animals**

Twenty white albino rats of weighing 150 g to 190 g were obtained from the College of Health Sciences, Benue State University, Makurdi, Nigeria. The animals were kept in polypropylene cages under room temperature, with 12-hour light and 12-hour dark cycle and were allowed to acclimatize for two weeks. The animals were provided commercial feed (Grand Cereals and Oil Mills Ltd, Bukuru, Jos, Nigeria) and clean water *ad libitum*. Protocols for this experiment was in accordance with the guidelines on the care and well being of research animals (14) and was approved by the Departmental Ethics Committee. 

The rats were paired overnight with sexually active males in the ratio of 2:1. Successful mating was confirmed by the presence of vaginal plug and or sperm cells in the vaginal smear the following morning between 9.00 and 10.00 hours. The day sperm cells were found in the vaginal smear was considered as day 1 of pregnancy. Thereafter, the female rats were randomly divided into four groups of five rats each thus: Group 1 received by gavage distilled water (1ml/ 100g) daily for 19 days and served as control. Groups II–IV rats were given the aqueous ethanolic extract of *Hymenocardia acida *by gavage at doses of 100, 200 and 400 mg/kg body weight daily for 19 days respectively. The rats were weighed daily and observed for any untoward effects.

On day 20 of gestation, each rat was laparatomised under high ether anaesthesia. The uterine horns were exteriorized and incised at the greater curvature of the horns. The latter were examined for sites of implantation and resorption. Number of corpora lutea of pregnancy, number of live foetuses as well as weights of the foetuses and placentae were also determined. The postcoitum fertility index was evaluated using the following parameters according to the methods of Tafessel *et al* (16) and Uchendu *et al* (1).

1. Percentage of pregnant female animals in each group (PPF)
2. Mean live foetal number per pregnant female (LFN)
3. Mean day 20 foetal crown-rump length (FCRL)
4. Mean corpus luteum number per pregnant female (CLN)

The fertility index (FI) of each group was calculated as

$\mathrm{FI}=\frac{\mathrm{LFN}\times \mathrm{FCRL}\times \mathrm{PPF}}{\mathrm{CLN}}$

The anti-implantation and antifertility activities of the extract were calculated as follows (16):

Anti-implantation activity= number of implants in control minus number of implants in test group divided by number of implants in control group multiplied by 100. Antifertility activity= number of rats showing no implantation divided by total number of animals multiplied by 100. 

**Statistical analysis**

Statistical evaluation of data was done using one–way analysis of variance (ANOVA). Means found to be significantly different at p<0.05 were separated using Duncan multiple range test. The results were expressed as mean± S.E.M. using Graph Pad Prism Version 3.0 for Windows (Graph Pad Software, San Diego, California).

**Declaration**

This is part of research work carried out by the first author under the supervision of second author. There is no conflict of interest. The work was funded by the first author.

## Results

The linear increases in maternal weights and weights gained (Table I) were higher in the control compared with the treatment groups. Oral administration of the extract from days 1 to 19 of gestation showed decrease (p< 0.05) in the number of corpora lutea of pregnancy and number of live foetuses (Table II). 

Weights of foetuses of extract treated female rats were also smaller (p<0.05) compared with the control. However, no gross morphological abnormalities were observed. All the females in the control group became pregnant and on laparatomy, were all found to have live foetuses. The extract did not cause any abortion or vaginal bleeding. The pregnant animals did not show signs of toxicity. All rats survived till the termination day. There were no foetal resorptions. 

Significant differences (p<0.05) in the mean foetal number per pregnant female (LFN), foetal crown-rump length (FCRL) and mean corpus luteum number per pregnant female (CLN) were observed in all the treated groups relative to the control (Table II). Antiimplantation activity of the treatment groups were 41.4%, 48.3% and 51.7% for groups II to IV respectively, whereas antifertility activity of the groups was found to be 40%, 60% and 60% in the same order (Table III). 

**Table I:** Effect of *Hymenocardia acida* stem bark extract on maternal and foetal weights. Values are expressed as means± S.E.M.; N= 5.

**Parameters**		**Control**	**100 mg/kg**	**200 mg/kg**	**400 mg/kg**
Maternal weights (g)					
	Day 1	151. 40 ± 1. 40	152. 40 ± 1. 96	152. 40 ± 1. 40	152. 00 ± 1. 99
	Day 7	162. 33 ± 2. 61^a^	159. 33 ± 3. 48^b^	152. 50 ± 3. 38^b^	152. 67 ± 3. 72^b^
	Day 14	180. 50 ± 2. 59^a^	164. 00 ± 3. 56^ab^	156. 33 ± 2. 61^b^	157. 75 ± 2. 66^b^
	Day 19	198. 75 ± 3. 68^a^	182. 00 ± 3. 00^ab^	166. 00 ± 2. 35^b^	165. 67 ± 2. 34^b^
Maternal weights (g)					
	Day 0 – 5	12. 60 ± 1. 80^a^	3. 00 ± 1.41^b^	3. 00 ± 1.18^b^	4. 33 ± 1.44^ab^
	Day 6 – 14	10. 80 ± 3. 92^a^	8. 40 ± 3. 80^ab^	8. 25 ± 3. 30^ab^	7. 20 ± 2. 06^b^
	Day 15 – 19	18. 80 ± 1. 71^a^	17. 67 ± 2. 29^a^	12. 20 ± 1. 12^ab^	7. 00 ± 1. 89^b^
	Day 0 – 19	45. 00 ± 2. 86^a^	37. 33 ± 3. 41^ab^	22. 25 ± 3. 31^b^	17. 50 ± 2. 51^b^
Foetal weight (g)		2. 69 ± 0. 21^a^	1. 82 ± 0. 40^ab^	1. 56 ± 0. 20^b^	1. 65 ± 0. 40^b^
Placental weight (g)		0. 47 ± 0. 02^a^	0. 27 ± 0. 01^ab^	0. 14 ± 0. 02^b^	0. 17 ± 0. 01^b^

Means with different superscripts in a row are significantly different (p=0.05). “N” represents number of animals used in each group.

**Table II:** Effect of *Hymenocardia acida* stem bark extract on foetal score. Values are expressed as means ± S.E.M; “N” = 5.

**Groups**	**No. of live foetuses(LFN)**	**No. of dead foetuses**	**REN**	**FCRL (cm)**	**CLN**	**PPL (%)**	**F.I.**
Control	7. 00 ± 0. 58^a^	0. 00 ± 0. 00	0.00 ± 0. 00	3. 56 ± 0. 21^a^	8. 40 ± 1. 03^a^	100^a^	990. 81
100 mg/kg	5. 67 ± 0.33^b^	0. 00 ± 0. 00	0.00 ± 0. 00	3. 50 ± 0. 31^ab^	5. 80 ± 1. 02^ab^	60^b^	486. 82
200 mg/kg	4. 50 ± 0. 50^ b^	0. 00 ± 0. 00	0. 00± 0. 00	2. 90 ± 0. 20^b^	5. 40 ±1. 34^ab^	40^c^	174. 00
400 mg/kg	3. 50 ± 0.50^ b^	0. 00 ± 0. 00	0. 00 ± 0. 00	2. 75 ± 0. 45^b^	4. 80 ± 1. 39^b^	40^c^	147. 64

Means with different superscripts in a row are significantly different (p= 0.05). “N” represents number of animals used in each group.

PPL- Percentage of Pregnant Females per Group; FCRl – Foetal Crown – Rump Length.

F.I. – Fertility; CLN – Corpus Luteum Number. LFN- Live foetal Number; REN – Resorbed Embryo Number.

**Table III:** Antifertility and antiimplantation activities of Hymenocardia acida stem bark extract

**Groups**	**No. pregnant/** **No. tested**	**No. of dead** **rats**	**No. of rats showing** **implantations**	**Total No. of** **implantations**	**Anti-implantation** **activity (%)**	**Anti-fertility** **activity (%)**
Control	5/5	0	5	29	Nil	Nil
100 mg/kg	5/3	0	3	17	41. 4	40. 0
200 mg/kg	5/2	0	2	9	48. 3	60. 0
400 mg/kg	5/2	0	2	7	51. 7	60. 0

## Discussion

Administration of *H. acida* extract at the test doses from days 1 to 19 of pregnancy resulted in strong antiimplantation and antifertility activities (Table III). At necropsy, no evidence of embryo resorption was found in the non pregnant rats. Several reports indicate antifertility activity of crude extracts (17, 18) and active compounds in animal models (19). Although there were neither deaths nor clinically observable, treatment-related inhibitory effects of *H. acida* on the pregnant rats, changes in maternal body weights (Table I) provide a good index of the integrity of maternal homeostasis (20). In the present study, significant decreases (p< 0.05) in maternal body weights were observed when aqueous ethanolic extract of *H. acida* stem bark was given to the rats. 

The low body weight gain in pregnant rats given the extract might suggest nil or few number of implantations. It is well established that the implantation index correlates with the number of corpora lutea and indicates blastocyst implantation in the endometrium (21) as well as normal reproductive capacity (22). *Hymenocardia acida* extract significantly reduced the number of implantations at the test doses (Table III). The mechanism of antiimplantation activity of *Monecha ciliatum* has been explained by its strong uterotonic property (23). Similarly, *Musanga cecropioides*, a common Nigerian medicinal plant used for its oxytocic effect has been reported to increase uterine contraction in a dose - dependent manner (24). 

In a pilot study, the aqueous ethanolic extract of *Hymenocardia acida* elicited contractile effect on the isolated uterine muscle tissue (unpublished observation). However, the mechanism of antiimplantation activity of the extract requires further investigation.

The rat endometrium is sensitive to blastocyst signals in the morning of day 5 (25), but can experience failure of blastocyst implantation due to hostile and incompetent uterine environment (26), antizygotic or blastocytotoxic property (27) and expulsion of embryo (28). Previous reports on antifertility effects of medicinal plants which showed resorptions of embryos and abortions (29) are not in agreement with the present study where we observed neither resorption sites nor gross malformations of the foetuses. *Hymenocardia acida* perhaps did not act as an abortifacient since there was no vaginal bleeding. However, there were reductions in foetal and placental weights of the treatment groups. 

The endometrial environment might not be conducive for implantation (30) as revealed by the smaller litter size. In addition to the endometrial microenvironment, hyper motility of the myometrium can also prevent implantation, especially in view of the effect of the extract on the isolated tissue (data not shown).

The reduction in foetal crown- rump length (FCRL) which is a parameter for foetal growth agrees with previous findings of impaired placental formation or placental insufficiency (31) and foetal development (32). A reduction in weights of foetuses of pregnant rats as observed in the present study had also been reported when *Acanthus montanus* leaves extract was administered to pregnant rats during gestation (33). However, other investigators showed that most pure compounds of *A*. *indica* were of relatively low reproductive toxicity compared with the crude seed oil (34). Similarly, aqueous extract of *Garcinia kola* administered to pregnant rats did not affect the number and weights of foetuses as well as implantation sites (35).

Previous reports indicate the presence of flavonoids (36), alkaloids (37) and terpenoids (1) in medicinal plants with contraceptive or pregnancy interceptory effects. In the present study, phytochemical screening of aqueous ethanolic extract of *H. acida* stem bark revealed the presence of alkaloids, tannins, flavonoids and terpenoids. 

Fertility of rats in groups treated with the extract was significantly different (p<0.05) from the control as reflected by the reduced pregnancy and fertility index (Table II). A similar observation on reduced fertility was made when pregnant rats were treated with *Dalbergia saxatilis* (1). 

The results suggest that aqueous ethanolic extract of *Hymenocardia acida* stem bark could induce inhibitory effects on reproductive functions in female albino rats.

## References

[1] Uchendu CN, Kamalu TN, Asuzu IU (2000). A preliminary evaluation of antifertility activity of a triterpenoid glycoside from Dalbergia saxatilis in female Wistar rats. Pharmacol Res.

[2] Srivastava S, Keshri G, Bhargevan B, Singh C, Singh MM (2007). Pregnancy interceptive activity of the roots of Calotropis gigantea Linn in rats. Contraception.

[3] Muller JC, Botelho GG, Bufalo AC, Boaereto AC, Rattman YD, Martins ES (2009). Morinda citrifolia Linn (Noni): In vivo and in vitro reproductive toxicology. JEthnopharmacol.

[4] Costa-Silva JH, Lima CR, Silva EJ, Araujo AV, Fraga MC, Ribeiro E Ribeiro A (2008). Acute and sub acute toxicity of the Carapa guianensis Aublet (Meliaceae) seed oil. J Ethnopharmacol.

[5] Muanza DN, Euler KL, Williams L, Newman DS (1995). Screening for antitumor and anti-HIV activities of nine medicinal plants from Zaire. Int J Pharmacol.

[6] Mpiana P T, Tshibanga DS, Shetonde OM, Ngbolua KN (2007). In vitro antidrepanocytary activity (antisickle cell anaemia) of some Congolese plants. Phytomedicine.

[7] Ukwe CV (2004). Evaluation of the anti-ulcer activity of aqueous stem bark extract of Hymenocardia acida. Nigerian Journal of Pharmaceutical Research.

[8] Tona L, Kambu K, Masia K, Cimanga R, Apers S, De bruyne T (1999). Biological screening of traditional preparations from some medicinal plants used as antidiarrhoeal in Kinshasha, Congo. Phytomedicine.

[9] Igoli JO, Ogeji OG, Tor-Anyiin TA, Igoli NP (2005). Traditional medical practice amongst the igede people of Nigeria. Afr J Trad CAM.

[10] Hoet S, Opperdoes F, Brun R, Adjakidje V, Quetinleclercq J (2004). In vitro antitrypanosomal activity of ethno pharmacologically selected Beninese plants. J Ethnopharmacol.

[11] Kamanzi Atindehook K, Schmid C, Brun R, Kone MW, Traore D (2004). Antitrypanosomal and antiplasmodial activity of medicinal plants from Cote d’Ivoire. J Ethnopharmacol.

[12] Vonthron-Senecheau C, Weniger B, Quattara M, Bi FT, Kamenan A, Lobstein A (2003). In vitro antiplasmodial activity and cytotoxicity of ethno botanically selected Ivorian plants. J Ethnopharmacol.

[13] Abu AH, Uchendu CN, Ofukwu RA (2009). In vitro antitrypanosomal activity of crude extracts of some Nigerian medicinal plants. J Appl Biosc.

[14] National Institute of Health (N.I.H) (1985). Guide for the care and use of laboratory animals.

[15] Wong PY, Lau SK, Fu WO (1987). Antifertility effects of some sulphonamides and related compounds and their accumulation in the rat epididymis. J Reprod Fertil.

[16] Tafessel G, Mekonnen Y, Makonnen E (2005). In vivo and in vitro antifertility and antiimplantation properties of Leonotis ocymifolia in rats. Afr J Trad CAM.

[17] Uchendu CN, Isek T (2008). Antifertility activity of aqueous ethanolic leaf extract of Spondias mombin (Anacardiaceae) in rats. Afr Health Sci.

[18] Yadav R, Jain GC (2009). Antifertility effect and hormonal profile of petroleum ether extract of seeds of Cassia fistula in female rats. International Journal of Pharmacology and Technology Research.

[19] Hikim AP, Lue YH, Wang C, Reutrakul V, Sangsuwan R, Swerdloff RS (2000). Post testicular antifertility action of triptolide in the male rat: evidence for severe impairment of cauda epididymal sperm ultra structure. J Androl.

[20] Almeida FC, Lemonica IP (2000). The toxic effects of Coleus barbatus on the different periods of pregnancy in rats. J Ethnopharmacol.

[21] Tafessel G, Mekonnen Y, Makonnen E (2006). Antifertility effect of aqueous and ethanol extracts of the leaves and roots of Asparagus africanus in rats. Afr Health Sci.

[22] Chang CV, Felicio AC, Reis JE, Guerra MdE O, Peters VM (2008). Fetal toxicity of Solanum lycocarpum (Solanaceae) in rats. J Ethnopharmacol.

[23] Uguru MO, Okwuasaba FK, Ekwenchi EE, Uguru VE (1998). Uterotonic properties of the methanolic extract of Monechma ciliatum. J Ethnopharmacol.

[24] Ayinde BA, Onwukaeme DN, Nworgu ZAM (2006). Oxytocic effects of the water extract of Musanga cecropioides R Brown (Moraceae) stem bark.. Afr J Biotechnol.

[25] Singh MM, Kamboj VP (1992). Fetal resorption in rats treated with an antiestrogen in relation to luteal phase nidatory estrogen secretion. Acta Endocrinol (Copenh).

[26] Emiliani S, Delbaere A, Devreker F, Englert Y (2005). Embryo-maternal interactive factors regulating the implantation process: Implications in assisted reproduction. Reprod Biomed Online.

[27] Hafez B, Hafez ESE (2000). Reproduction in Farm Animals.

[28] Orihuela PA, Rios M, Croxatto HB (2001). Disparate effects of estradiol on egg transport and oviductal protein synthesis in mated and cyclic rats. Biol Reprod.

[29] Vasudeva N, Sharma SK (2007). Estrogenic and pregnancy interceptory effects of Achyranthes aspera Linn root. Afr J Trad, Complement Altern Med.

[30] Hazarika A, Sarma HN (2007). Effects of crude root extract of Polygonum hydropiper on estrous cycle and induction of reversible sterility in female albino rat. Journal of Endocrinology and Reproduction.

[31] Rasheed RA, Bashir AK, Ali BH, Padmanabhan R (1997). Effect of Rhazya stricta on the developing rat fetus. Reprod Toxicol.

[32] Schwarz A, Pinto E, Haraguchi M, de Oliveira CA, Bernardi MM, Spinosa HS (2007). Phytochemical study of Solanum lycocarpum unripe fruit and its effects on rat gestation. Phytotherapy Research.

[33] Nana P, Asongalem ER, Foyet HS, Folefoe GN, Dimo T, Kamtchouing P (2008). Maternal and developmental toxicity evaluation of Acanthus montanus leaves extract administered orally to Wistar pregnant rats during organogenesis. J Ethnopharmacol.

[34] Boeke SJ, Boersma MG, Alink GM, Van loon JJA, VanHuis A, Dicke M (2004). Safety evaluation of neem (Azadirchta indica) derived pesticides. J Ethnopharmacol.

[35] Iranloye B, Owokunle B (2008). Effect of Garcinia kola seed extract on female reproductive functions in rats. Int J Pharmacol.

[36] Hiremath SP, Rudresh K, Badami S, Patil SB, Patil RS (1999). Post-coital antifertility activity of Acalypha indica L. J Ethnopharmacol.

[37] Ravichandra V, Suresh B, Satishkumar MN, Elango K, Srinivasan R (2007). Antifertility activity of hydroalcoholic extract of Ailanthus excelsa (Roxb): An ethno medicine used by tribes of Nilgiris region in Tamilnadu. J Ethnopharmacol.

